# Cyclic *trans*-phosphorylation in a homodimer as the predominant mechanism of EGFRvIII action and regulation

**DOI:** 10.18632/oncotarget.24058

**Published:** 2018-01-06

**Authors:** Wojciech Stec, Kamila Rosiak, Cezary Treda, Maciej Smolarz, Joanna Peciak, Marcin Pacholczyk, Anna Lenart, Dawid Grzela, Ewelina Stoczynska-Fidelus, Piotr Rieske

**Affiliations:** ^1^ Research and Development Unit, Celther Polska Ltd., Lodz, Poland; ^2^ Department of Tumor Biology, Medical University of Lodz, Lodz, Poland; ^3^ Institute of Automatic Control, Silesian University of Technology, Gliwice, Poland; ^4^ Research and Development Unit, Personather Ltd., Lodz, Poland

**Keywords:** EGFR, EGFRvIII, dimerization, phosphorylation

## Abstract

Despite intensive research no therapies targeted against the oncogenic EGFRvIII are present in the clinic. One of the reasons is the elusive nature of the molecular structure and activity of the truncated receptor. The recent publications indicate the EGF-bound wild-type EGFR to *trans*-phosphorylate the EGFRvIII initiating aberrant signaling cascade. The elevated stability of the mutant receptor contributes towards oncogenic potential, preventing termination of signaling by receptor degradation. Here, we show that inhibition of phosphatases leads to a marked increase in phosphorylation of wild-type EGFR and EGFRvIII, indicating that both undergo cyclic rounds of phosphorylation and dephosphorylation on all investigated tyrosine residues, including Tyr1045. Still, we observe elevated stability of the mutant receptor, suggesting phosphorylation as insufficient to cause degradation. Hyperphosphorylation of EGFRvIII was hindered only by EGFR tyrosine kinase inhibitors. Co-immunoprecipitation as well as semi-native Western blotting structural analyses together with functional investigation of EGFRvIII’s phosphorylation following depletion of wild-type EGFR by shRNA or EGF-mediated degradation indicated homodimerization as the predominant quaternary structure of the mutant receptor. Dimers were observed only under non-reducing conditions, suggesting that homodimerization is mediated by covalent bonds. Previous reports indicated cysteine at position 16 to mediate covalent homodimerization. Upon its substitution to serine, we have observed impaired formation of dimers and lower phosphorylation levels of the mutated oncogene. Based on the obtained results we propose that EGFRvIII is predominantly regulated dynamically by phosphatases that counteract the process of *trans*-phosphorylation occurring within the homodimers.

## INTRODUCTION

Glioblastoma (GB) is the most common primary brain tumor, characterized by a very high mortality rate. The current first line treatment, a combination of neurosurgery with adjuvant radio- and chemotherapy, extends patients life on average from 4 to 14 months [1]. Approximately 50% of GBs is characterized by overexpression of the epidermal growth factor receptor (EGFR) and about half of those is positive for a mutant version of EGFR, termed variant III or vIII, for short (EGFRvIII) [2]. Besides GBs, also cancers of head and neck, breast, prostate, intestines and non-small cell lung cancer have been previously described to express this truncated oncogene, although at lower prevalence and to a smaller extent [3]. EGFRvIII arises from a deletion of exons 2–7 from the wild-type gene, which encodes the portion of the extracellular domain of the receptor that is responsible for ligand binding. The resulting protein is present in the plasma membrane, where it is highly stable with protein half-life extending much beyond that of the wild-type protein [4, 5]. The underlying reasons of this elevated stability remain cryptic with conflicting reports on the role of protein phosphorylation in the degradation process published previously [6–8].

Despite intensified research on the structure-function relationship in the context of dimerization and ligand binding by wild-type EGFR (EGFRwt), our understanding of the receptor activation mechanism remains incomplete. This is also true for EGFRvIII protein complex formation and dynamics. Several reports focused on the state in which EGFRvIII is present in the plasma membrane. Chu and colleagues indicated that EGFRvIII is present only as monomers [9]. In contrast, several publications have provided contradicting results indicating EGFRvIII to form dimers, including homodimers [10–14]. Ymer et al., has shown that by altering the sequence of EGFRvIII gene by substitution of the free cysteine at position 16 to serine, formation of stable covalently linked homodimers was impaired [15]. On the other hand, Bogler lab provided evidence that forcing EGFRvIII to homodimerize resulted in elevated aberrant signaling, indicating that homodimerization under native conditions occurs to a limited extent [10]. Others have indicated heterodimers with wild-type EGFR or other receptor tyrosine kinases (RTKs) to be the predominant form of EGFRvIII presence in the plasma membrane [11, 13, 14, 16–18]. Besides composition of the receptor complex, also the symmetry of interaction was investigated. Using a series of single-point mutated proteins Fan and colleagues demonstrated that EGFRvIII is a donor (substrate) for EGF-activated EGFRwt (acceptor), but the opposite is not true [16]. Using the same approach, Kancha and colleagues indicated that EGFRvIII can asymmetrically phosphorylate ErbB3, while others indicated EGFRvIII to catalyze phosphorylation of MET [11, 17, 18].

In this study, we show that EGFRvIII is constitutively active as determined by elevated phosphorylation status compared to the wild-type receptor in the absence of ligand stimulation. This phosphorylation pattern is likely to be a convergence of the kinase activity and regulation by phosphatases, as determined by elevated phosphorylation caused by treatment with pan-phosphatase inhibitors. As EGFR monomers undergo trans-phosphorylation, we exploit phosphorylation status as a readout to study structural and functional aspects of the EGFRvIII dimerization. Using EGFR kinase inhibitors as well as manipulation of the protein structure and expression levels, we determine that EGFRvIII undergoes phosphorylation in a homodimer with no interactions with the wild-type receptor observed. Furthermore, our data indicate that EGFRvIII is prone to generating stable covalent dimers via Cys16, however, a population of transient complexes is likely to exist as well.

## RESULTS

### Differential phosphorylation status of EGFRvIII and EGFRwt

**Figure 1:**
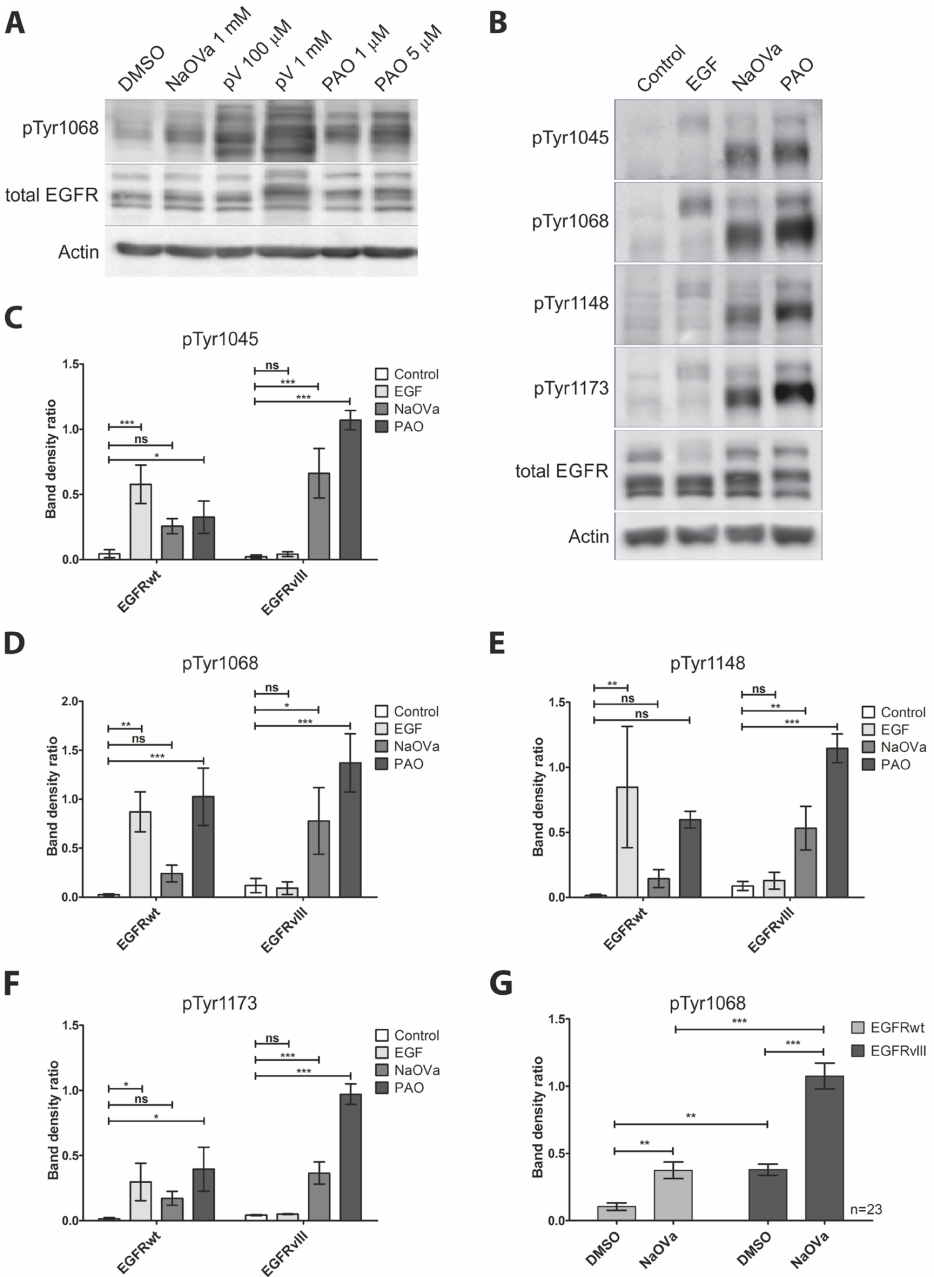
Treatment of DK-MG cells with phosphatase inhibitors results in hyperphosphorylation of EGFRwt and EGFRvIII on majority of tyrosine residues (**A**) Western blot analysis of EGFR phosphorylation in cells treated with NaOVa, pV or PAO at indicated concentrations for 1 h. (**B**) Analysis of phosphorylation status of EGFR on selected tyrosine residues following stimulation with 20 ng/mL EGF, 1 mM NaOVa or 0.5 µM PAO. (**C–F**) Ratio of phosphorylated tyrosine to the total EGFR protein for the wild-type and mutant protein following stimulation as indicated in B, for residue Tyr 1045 (C), Tyr1068 (D), Tyr1148 (E) and Tyr 1173 (F). Statistical analysis performed using one-way ANOVA with post-analysis Bonferroni’s multiple comparisons test for each residue within one receptor variant. (**G**) Quantification of phosphorylated tyrosine 1068 to total EGFR for EGFRwt and EGFRvIII is presented. Statistical analysis performed using two-way ANOVA with post-analysis Bonferroni’s multiple comparisons test, *n* = 23. ^***^p < 0.005; ^**^p < 0.01;^*^p < 0.05; ns – not significant. Error bars indicate SEM. Immunoblots have been uniformly adjusted for brightness and contrast to facilitate interpretation.

Previous reports on the activity of EGFRvIII described it as a constitutively active kinase [5, 7, 12, 19–21]. To investigate the dynamics of EGFRvIII phosphorylation, we have utilized a selection of protein tyrosine phosphatase (PTP) inhibitors, namely sodium orthovanadate (NaOVa), pervanadate (pV) and phenylarsine oxide (PAO) [22]. To allow for comparison with the wild-type receptor that was reported to be regulated by PTPs [23–27], we utilized previously described by us DK-MG^high^ cells, which endogenously express full-length and truncated form of EGFR [28]. To avoid the issue of putative receptor stimulation by exogenous ligands, we have treated cells with cell-permeable PTP inhibitors in serum-free media. Phosphorylation of EGFRwt and EGFRvIII was analyzed by Western blotting, taking advantage of the difference in the molecular size of the two proteins (170 kDa and 135 kDa, respectively) (Figure 1A). Compared to the control cells, all three inhibitors increased phosphorylation levels of Tyr1068 on both EGFR variants, however, treatment with pV produced a number of additional bands, making the outcome difficult to interpret and therefore we did not use this chemical in subsequent experiments. The effects of NaOVa and PAO on protein phosphorylation were both, time- and dose-dependent, suggesting it to be a result of enzymatic activity (Supplementary Figure 1A and 1B, respectively). Both compounds displayed toxic effects on cells at high concentrations or upon prolonged incubation, therefore, we have selected 1 mM NaOVa and 0.5 µM PAO as optimal concentrations with 1 hour of incubation for further testing.

To investigate the effect of both PTP inhibitors in greater detail, we have utilized a panel of antibodies specific to phosphorylated tyrosines (Figure 1B). To address the issue that ligand-dependent and ligand-independent activation of the receptor tyrosine kinases can affect their stability we have utilized densitometry measurements, results of which are expressed as a ratio of phosphorylated to total protein for each tyrosine residue (Figure 1C–1F). As expected, stimulation with EGF resulted in a significant increase in phosphorylation on all tyrosines of the wild-type receptor but not of the mutant variant. Both PTP inhibitors caused a prominent increase in phosphorylation levels of EGFRvIII on all residues examined, including Tyr1045. Interestingly, hypophosphorylation of this tyrosine has been previously reported on a couple of occasions and linked to the elevated stability of the protein [7, 8]. Indeed, we have not observed any degradation of the truncated receptor even in the presence of cycloheximide to prevent de novo protein synthesis (Supplementary Figure 2).

Statistical analysis indicated that the shift in phosphorylation of individual tyrosines was not always significant in case of the wild-type receptor, which might be a result of a combination of limited sensitivity of the method and a relatively small number of replicas (*n* = 3). To test this hypothesis, we have focused on NaOVa, a less potent PTP inhibitor of the two, and analyzed its effect on phosphorylation of Tyr1068, considered crucial for EGFR signaling, on significant number of immunoblots (*n* = 23, Figure 1G). Statistical analysis of the larger sample pool clearly indicated that NaOVa efficiently increased phosphorylation levels of both EGFR versions. Considering the less potent nature of NaOVa, it is safe to assume that the same is likely to be true for the more potent PAO. Furthermore, the phosphorylation level of EGFRvIII under control conditions (DMSO) was determined to be higher compared to the EGFRwt, confirming the mutant receptor as constitutively active [5, 7, 12, 19–21]. Following treatment with NaOVa, phosphorylation level of both proteins was increased with marked difference between the wild-type and mutant version.

Finally, we assayed the effect of PTP inhibitor treatment on EGFRvIII phosphorylation on primary tumor cells derived from glioblastoma resection (Supplementary Figure 3). In line with previous results, stimulation with 20 ng/mL of EGF increased phosphorylation of the wild-type, but not the mutant receptor. In contrast, treatment with NaOVa resulted in elevated phosphorylation of EGFRvIII and EGFRwt, confirming effect of PTP inhibitor as not an artifact of the stable cell line.

### Ligand binding to the EGFRwt does not affect phosphorylation of EGFRvIII

**Figure 2:**
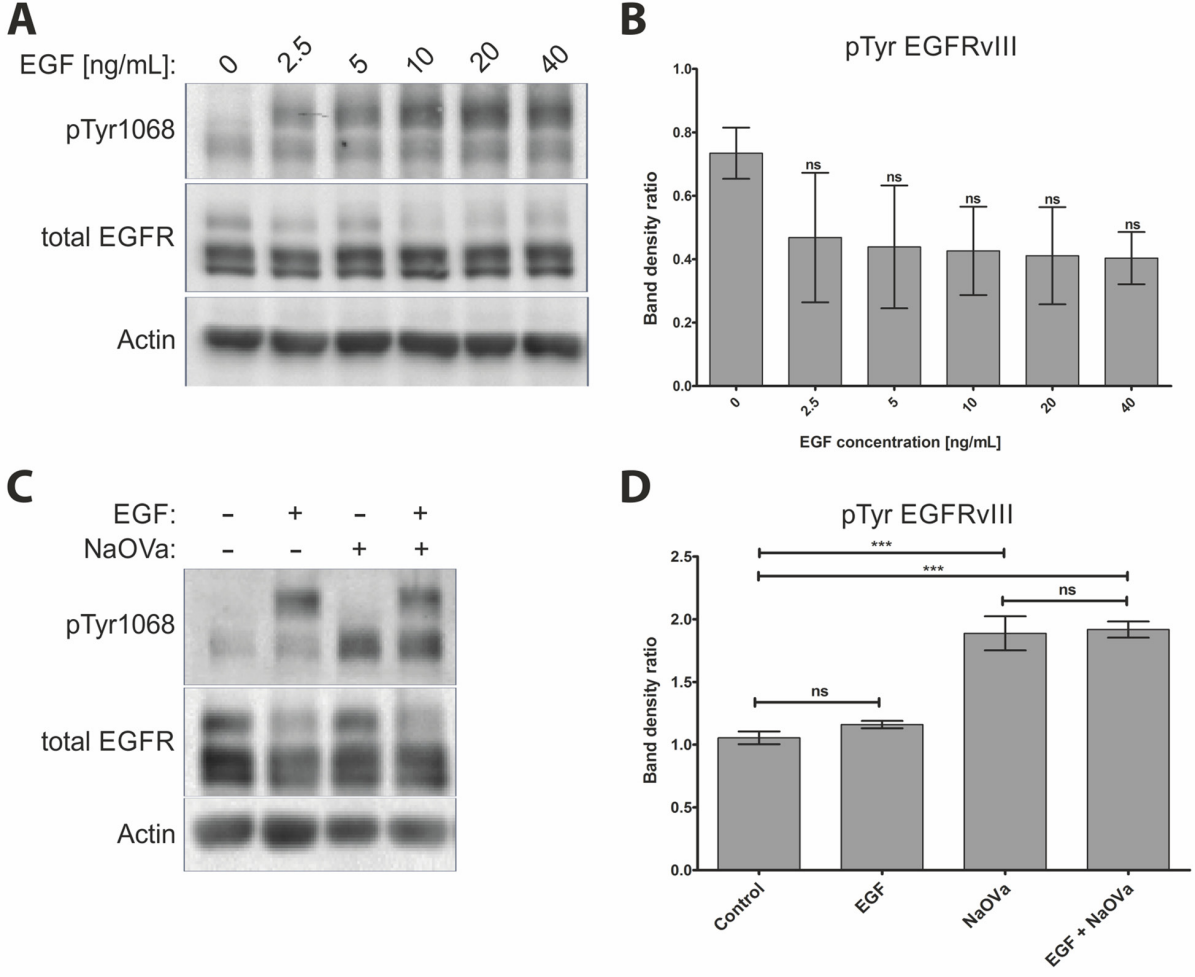
Treatment with EGF does not affect phosphorylation of EGFRvIII (**A**) DK-MG cells treated with indicated concentration of EGF for 10 min were lysed and analysed by Western blotting. (**B**) Quantification of EGFRvIII phosphorylation as shown in A. Statistical analysis performed using one-way ANOVA with post-analysis Bonferroni’s multiple comparisons test. (**C**) Western blot analysis of cells stimulated for 1 h with 20 ng/mL EGF, 1 mM NaOVa, concomitant EGF and NaOVa or Control cells, as indicated. (**D**) Quantification of blots as shown in C, with normalization to wild-type receptor under Control conditions. Statistical analysis performed using two-way ANOVA with post-analysis Bonferroni’s multiple comparisons test. ^***^p < 0.005; ns – not significant; n of at least 3 experiments. Error bars indicate SEM. Asterisks indicate comparison to control cells or as indicated by the bars beneath them. Immunoblots have been uniformly adjusted for brightness and contrast to facilitate interpretation.

Stimulation of the DK-MG^high^ or primary tumor cells with EGF did not result in elevated phosphorylation of the mutant receptor, despite the marked increase in phosphorylation of the wild-type EGFR (Figure 1B–1F, Supplementary Figure 3), which is in contrast to previous reports [11, 14, 16, 29]. Investigation of the effect of varying concentrations of the ligand on phosphorylation status of the mutated receptor indicated no relationship between the two (Figure 2A, quantified in B). Phosphorylation of EGFRwt was evident already following exposure to 2.5 ng/mL of ligand and was increased by higher concentration until it reached a plateau above 10 ng/mL, with approximately 20 ng/mL considered as physiological concentration [30]. Increasing length of stimulation to two hours at relatively high concentration of EGF did not have any impact either (Supplementary Figure 4). Considering that phosphorylation and dephosphorylation are subsequent processes, we wanted to investigate potential additive or synergistic effects between exposition to EGF and NaOVa. Concomitant treatment of cells with the ligand and pan-phosphatase inhibitor did not elevate phosphorylation levels of EGFRvIII beyond the levels observed for NaOVa treatment on its own (Figure 2C, quantified in D). Taken together, our data indicate that EGF stimulation is dispensable for cyclic phosphorylation of EGFRvIII.

### Trans-phosphorylation of EGFRvIII is not mediated by the wild-type receptor

**Figure 3:**
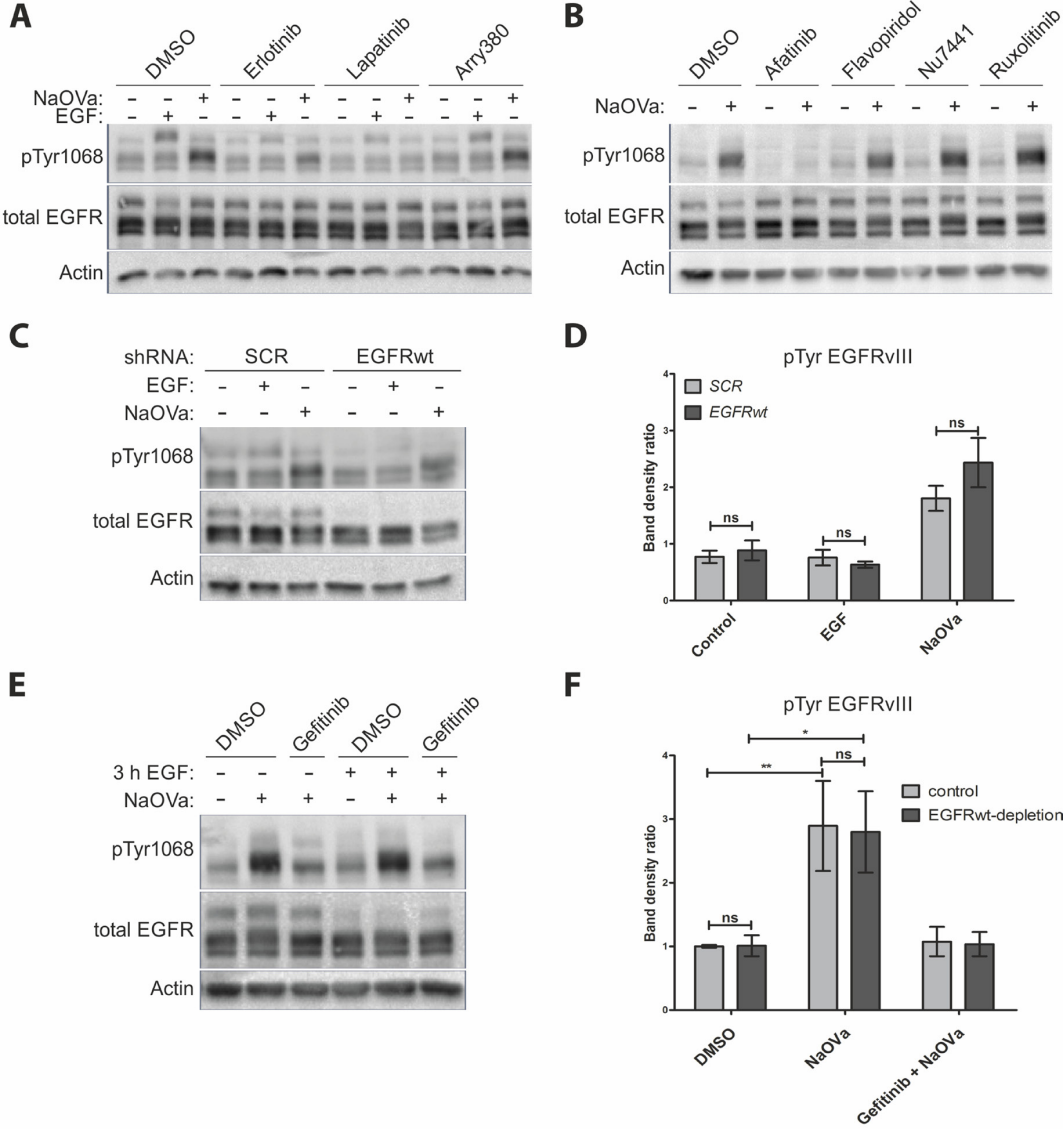
EGFRvIII is not phosphorylated by EGFRwt (**A**) Western blot analysis of cells treated with indicated inhibitors prior to and concurrently with EGF or NaOVa treatment for 1 h. Concentrations of inhibitors were based on literature reports. (**B**) Cells were treated with inhibitors specific to pan-JAK (ruxolitinib), CDK2 (flavopiridol) or DNA-PK (Nu-7441) prior to and concurrently with NaOVa treatment. Concentrations of inhibitors were based on literature reports. (**C**) Cells transiently expressing control scrambled (SCR) shRNA or shRNA targeted against EGFRwt were treated as indicated and analyzed by Western blotting. (**D**) Quantification of blots as shown in C, with columns representing ratio of phosphorylated to total EGFRvIII protein. (**E**) Cells treated with cycloheximide and stimulated with EGF for 3 h as indicated. Where indicated, cells were incubated for another 30 min with DMSO or gefitinib prior to 1 h treatment with NaOVa. (**F**) Quantification of blots as in E for EGFRvIII, with normalization to DMSO treated Control cells. Statistical analysis performed using two-way ANOVA with post-analysis Bonferroni’s multiple comparisons test. ^**^p < 0.01; ^*^p < 0.05; ns – not significant; n of at least 3 experiments. Error bars indicate SEM. Immunoblots have been uniformly adjusted for brightness and contrast to facilitate interpretation.

A number of reports has indicated trans-phosphorylation of EGFRvIII as a mechanism governing its activity. To identify the kinase responsible, we have pre-treated cells with erlotinib (EGFR specific inhibitor), lapatinib (inhibitor specific to EGFR and HER2) or arry-380 (HER2 specific) prior to stimulation with EGF or NaOVa (Figure 3A) [31, 32]. The former two inhibitors were capable of hindering phosphorylation of the EGFRwt following stimulation with the ligand and severely limited the increase in EGFRvIII phosphorylation levels upon NaOVa treatment. Arry-380 did not block phosphorylation of either protein irrespective of the stimulation method, suggesting that ErbB2 is not responsible for phosphorylation of the mutant receptor. Unfortunately, no inhibitors specific only to ErbB3 or ErbB4 exist, therefore we could not investigate role of those receptors separately. Erlotinib was also capable of preventing phosphorylation of EGFRwt and EGFRvIII in response to PAO stimulation, but not pV stimulation, confirming differences in activity between phosphatase inhibitors (Supplementary Figure 5). Use of selected inhibitors specific to other kinases reported to interact with EGFRvIII did not prevent mutant receptor’s phosphorylation following orthovanadate treatment, in contrast to afatinib, a pan-ErbB inhibitor, which effectively blocked phosphorylation (Figure 3B) [33–35]. The inhibitory role of erlotinib, gefitinib (working in an analogous fashion to erlotinib) and afatinib in preventing NaOVa-mediated hyperphosphorylation of EGFRvIII was confirmed on U87MG cells ectopically expressing mutant receptor (Supplementary Figure 6) [36]. Taken together, those data imply that EGFRvIII is a substrate of an EGFR kinase-containing protein.

To determine the identity of the kinase in question, we have utilized two approaches to deplete the wild-type receptor from the DK-MG^high^ cells. Firstly, we have transiently transduced cells with lentivirus containing shRNA targeting the EGFRwt mRNA or a scrambled control (Figure 3C). There were no differences observed in EGFRvIII phosphorylation levels between control and EGFRwt-depleted cells, despite the confirmation of the shRNA-mediated knockdown effectiveness at 80% on the mRNA level (Figure 3D and Supplementary Figure 7). Secondly, we have utilized the naturally occurring ligand-mediated degradation process, described for the EGFR, to deplete EGFRwt from the plasma membrane, whilst simultaneously preventing de novo protein synthesis with cycloheximide [37–39]. Treatment with NaOVa strongly increased phosphorylation level of the EGFRvIII, irrelevant of previous treatment with EGF (Figure 3E, quantified in F), which caused a marked decrease in the blotted EGFRwt protein levels as well as intracellular puncta reminiscent of endocytosed protein upon immunocytochemical analysis (Supplementary Figure 8). Gefitinib effectively suppressed the effect of NaOVa treatment on mutant receptor’s phosphorylation, suggesting that even upon decreased levels of EGFRwt, the remaining EGFR kinase in the form of EGFRvIII is responsible for EGFRvIII hyperphosphorylation (Figure 3E and 3F). Taken together, our data indicate that the wild-type receptor is dispensable for EGFRvIII phosphorylation, leaving trans-phosphorylation in a homodimer as the most viable possibility.

**Figure 4:**
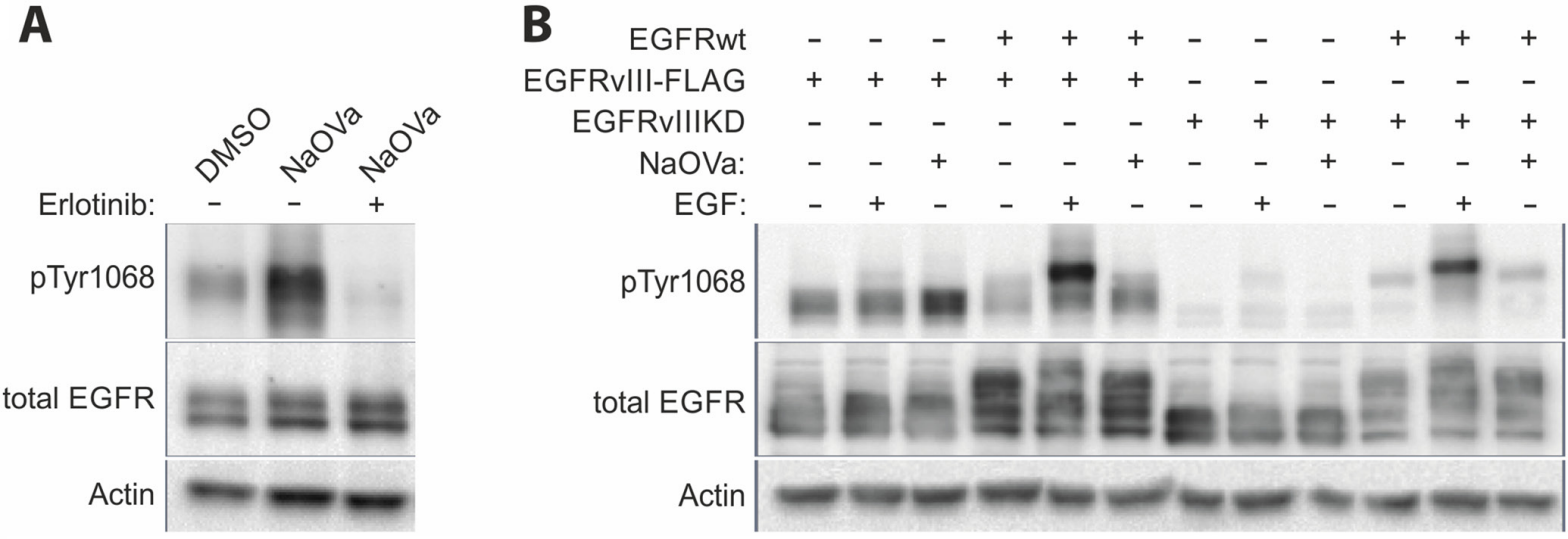
EGFRwt is dispensable for EGFRvIII phosphorylation (**A**) AD293 cell lines stably expressing EGFRvIII was treated with erlotinib prior to and simultaneously with NaOVa stimulation. (**B**) AD293 cell line expressing either naïve or kinase dead (KD) version of EGFRvIII on its own or together with EGFRwt have been treated with EGF or NaOVa, as indicated.

The strong co-expression of the wild-type as well as mutant receptors in the DK-MG line complicates analysis of the protein-protein interactions. To facilitate the analysis, we have utilized AD293 cell line that endogenously displays low levels of the EGFRwt expression (approximately 7-fold lower than in DK-MG; Supplementary Figure 9) to establish stable cell lines constitutively expressing EGFRvIII (with or without FLAG-tag) on its own or together with the wild-type receptor overexpression. The strong phosphorylation of EGFRvIII observed in response to NaOVa treatment was not hindered by the low levels of the EGFRwt (Figure 4A). Erlotinib efficiently suppressed the effects of the orthovanadate, confirming previous results obtained on other cell lines (Figure 3A and Supplementary Figure 6). Taking advantage of the low endogenous expression of the EGFRwt, we have attempted to compare phosphorylation of the EGFRvIII in cell line that overexpresses the mutant receptor on its own or together with the wild-type EGFR (Figure 4B). Under the control conditions, phosphorylation status of EGFRvIII was comparable and did not change following stimulation with EGF. The increase in phosphorylation level following NaOVa treatment was not altered by EGFRwt overexpression, suggesting that homodimer rather than heterodimer with EGFRwt is required for phosphorylation of the mutant receptor.

To investigate such possibility, we have conducted an analogous experiment using a cell line expressing a kinase-dead (KD) variant of EGFRvIII, which carries a single point mutation (K478A, mutation analogous to K721A in the wild-type receptor) in the ATP-binding pocket that renders the kinase inactive (Figure 4B) [5, 40]. Importantly, the EGFRvIIIKD variant’s phosphorylation status under the control conditions was markedly lower than that of the naïve mutant’s and was not altered by addition of NaOVa, suggesting the critical role of the kinase domain of the EGFRvIII in the constitutive phosphorylation process. Levels of the expressed constructs were comparable, therefore protein stoichiometry is unlikely to explain differences observed between naïve EGFRvIII and KD version (Supplementary Figure 10). Overexpression of the EGFRwt did not have any effect on this process, irrespective of ligand-mediated stimulation, which did increase phosphorylation of the wild-type receptor. Additional approach at studying putative interaction between the wild-type receptor and FLAG-tagged EGFRvIII by the means of co-immunoprecipitation did not produce any band for the wild-type receptor (Supplementary Figure 11).

### EGFRvIII forms covalent homodimers via free cysteine

**Figure 5:**
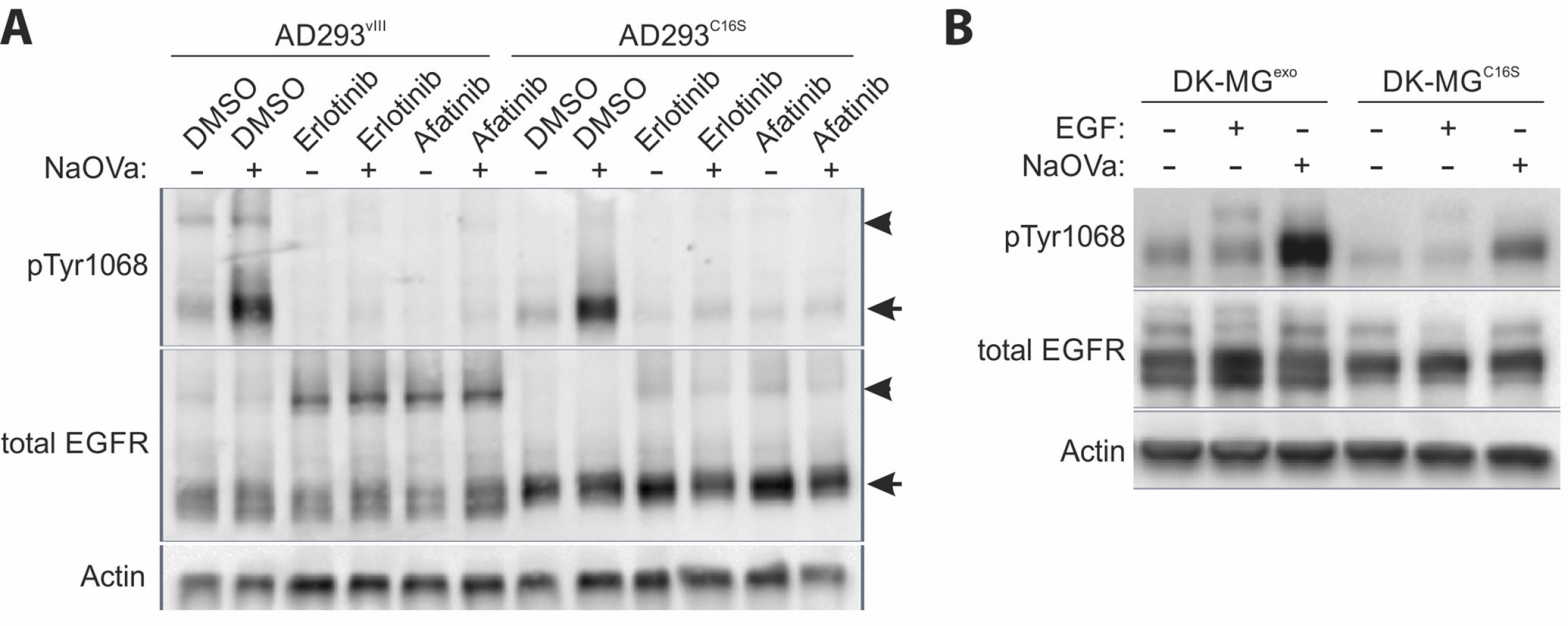
Homodimerization of EGFRvIII (**A**) Semi-native Western blot (non-reducing conditions) analysis of the AD293 cell lines expressing EGFRvIII or EGFRvIIIC16S treated simultaneously with EGFR TKIs and NaOVa, as indicated. Arrowheads indicate dimers, arrows point to monomers. (**B**) DK-MG^low^, cell line with marginal endogenous EGFRvIII expression, were transduced to constitutively express either naïve EGFRvIII (DK-MG^exo^) or C16S mutated version (DK-MG^C16S^). Cells were treated with NaOVa or EGF, as indicated. Immunoblots have been uniformly adjusted for brightness and contrast to facilitate interpretation.

As previous reports have indicated the cysteine at position 16 of the mutant receptor to be free, we have investigated whether it might be involved in formation of covalent dimers [11, 15]. To assess this possibility, we have analyzed capability of naïve EGFRvIII and EGFRvIII carrying C16S substitution to form dimers by semi-native Western blotting technique. By exclusion of the reducing agent from sample preparation this technique allows for investigation of covalently bound protein complexes based on the molecular size of the structure. Upon investigation of lysates from AD293 cells expressing naïve EGFRvIII, antibody recognizing shared EGFR epitope indicated presence of a protein complex at 270 kDa, corresponding in size to a putative EGFRvIII dimer, as well as monomerized protein at 135 kDa (Figure 5A). Treatment with NaOVa resulted in elevated phosphorylation of both versions of the mutant receptor, however, the ratio of phosphorylated dimers to monomers was much higher for the naïve protein, with majority of phosphorylated protein in the monomerized form for the C16S version. Interestingly, incubation with inhibitors of EGFR kinase resulted in elevated dimerization with simultaneous decrease in receptor phosphorylation levels for naïve and C16S variants. This observation is analogous to the effect of TKIs on EGFRwt receptor dimerization that has been previously reported by a number of groups [41, 42].

This observation has been confirmed using reducing Western blotting on the DK-MG^low^ line, characterized by much smaller fraction of EGFRvIII-positive cells, exogenously expressing either naïve EGFRvIII or the C16S variant [28]. The latter phosphorylated to much lower extent, when compared to the naïve protein, suggesting that the investigated residue must be an important component of the interaction interface (Figure 5B).

## DISCUSSION

The aspect of EGFRvIII dimerization was controversial ever since first characterization of the mutant receptor [2, 9, 13, 14]. The most recent publications in the field trend towards the critical role of the wild-type receptor bound by EGF that plays the role of the acceptor in activation of EGFRvIII (the donor) [14, 16]. This one-time phosphorylation is described as sufficient to activate aberrant signaling of the mutant receptor. However, our data suggest that EGFRvIII is predominantly trans-phosphorylated in a homodimer. First of all, we have not observed any effect of manipulation of the EGFRwt levels on EGFRvIII hyperphosphorylation following NaOVa treatment in any model investigated. Depletion of the wild-type receptor from DK-MG cells by the means of shRNA or ligand-mediated degradation did not affect EGFRvIII phosphorylation status (Figure 3). Overexpression of the wild-type receptor levels in AD293 cells, characterized by low endogenous levels of the receptor, did not have any effect either (Figure 4 and Supplementary Figure 9). Furthermore, stimulation of cells with EGF induced phosphorylation of the wild-type receptor that did not translate into phosphorylation of the truncated version on any line tested, irrespective of ligand dosage or length of stimulation (Figures 1–2 and Supplementary Figures (3–4). This effect was confirmed also by the use of the kinase-dead variant of the mutant receptor (Figure 4B). Considering that the naïve EGFRvIII becomes phosphorylated in response to NaOVa treatment, whilst EGFRvIIIKD does not, and EGFRwt does not play any role in the process, it can be concluded that EGFRvIII is solely responsible for its phosphorylation (Figure 4B). Concomitant stimulation of cells with EGF and NaOVa did not have any effect either (Figure 2C and 2D). In all instances however, the specific inhibitors of the EGFR kinase, but not of other kinases, were effective at hindering phosphorylation of the EGFRvIII in response to NaOVa as well as PAO treatments (Figure 3 and Supplementary Figure 5). Taken together, these data suggest that EGFRwt does not affect EGFRvIII’s phosphorylation. Those findings contrast previous reports indicating heterodimerization as the predominant means of EGFRvIII activation [5, 11, 14–16, 18, 43, 44].

Secondly, previous reports on the structure-function relationship of EGFRvIII have indicated that the deletion of the protein fragment from the extracellular domain has generated a free cysteine at position 16, that is highly reactive [11, 45]. The sensitivity of the putative dimers to β-ME suggests that C16 might be involved in formation of covalent bonds within the dimer (Figure 5A). In the semi-native Western blotting approach, we have shown that the ratio of dimers to monomers was severely decreased in case of the C16S mutant in comparison to the naïve protein. At the same time, we have not observed any significant bands that might imply heterodimerization and the co-immunoprecipitation study did not indicate any interaction between the wild-type and mutant EGFR (Supplementary Figure 11). It should be noted that recent reports indicate that EGFRwt undergoes monomerization prior to internalization, suggesting that heterodimeric interaction might be too brief to capture by the means of co-IP [46]. Still, the increase in phosphorylation levels of C16S variant following NaOVa treatment suggests that some interaction between two monomers, however brief and unstable, must occur to allow for trans-phosphorylation, a motion suggested in previous reports (Figure 5) [5, 14, 15, 20]. Such possibility could explain the reports on elevated carcinogenicity observed upon forced dimerization of EGFRvIII [47]. Moreover, the comparable effect of the EGFR tyrosine kinase inhibitors on dimerization of EGFRvIII and the C16S mutant is in line with previous reports on this phenomenon observed with the wild-type receptor [41, 42]. This suggests that the effect of the kinase domain on the dimerization process remains intact in the truncated proteins. Further investigation of the structure-function relationship of the mutant receptor as well as its quaternary structure presents interesting opportunity to gain mechanical understanding of the mechanism governing oncogene’s activity.

Furthermore, EGFRvIII is commonly described as highly stable in the plasma membrane, however, the underlying reasons remain controversial, with some reports indicating lack of phosphorylation of Tyr1045 or inability to interact with Cbl as the primary reasons [6–8]. Investigation of Tyr1045 phosphorylation in response to NaOVa or PAO treatment indicated that this residue is actively phosphorylated and dephosphorylated on EGFRvIII (Figure 1B and 1C). Despite phosphorylation of Tyr1045, we have confirmed the high stability of the mutant receptor in the DK-MG^high^ line, with no degradation observed even following 24 h incubation with cycloheximide (Supplementary Figure 2). Interestingly, stimulation with NaOVa did not obstruct ligand-mediated degradation of the full-length receptor (compare lane 2 and 4 in Figure 2C), suggesting that phosphorylation of the mutant receptor on Tyr1045 is insufficient to cause degradation of EGFRvIII. This is in line with previous reports suggesting presence of the cryptic internalization motifs in the EGFRwt that become displayed following ligand binding [33–35, 37, 38]. Our data imply that such motifs might not be displayed in EGFRvIII, contributing towards its elevated stability. Taking into consideration that endocytic process actively regulates quantity and quality of EGFRwt signaling as well as ultimately terminates it, it is plausible to assume that lack of degradation of EGFRvIII might be a crucial contributor towards its oncogenicity [48–50].

The phosphorylation status of EGFRvIII on Tyr1068 was elevated compared to EGFRwt when no external stimulant (ligand or otherwise) was present (Figure 1G). This result is in line with the previous reports suggesting the mutant receptor to be constitutively active [5, 7, 12, 19–21]. We do note however, that reaching such conclusion in a statistically significant manner required a large number of experiments reflecting the limitations of the model system as well as the method used. Despite our best attempts, we cannot exclude the possibility that DK-MG cells express EGFR ligands at low levels, antibodies are not completely specific to phosphorylated residues or the specificity of the signal was not sufficiently distinct from the background noise. Furthermore, comparisons of the phosphorylation status of EGFRwt and EGFRvIII under physiological conditions in glioma cells are likely to reveal different pattern due to the putative cross-talk between different pathways as well as presence of ligands acting in an auto- or paracrine manner [44, 51, 52]. Still, treatment with the two PTP inhibitors produced a clear increase in phosphorylation levels for both EGFR protein variants on all models assessed, with EGFRvIII’s phosphorylation being more prominent (Figures 1, 4 and Supplementary Figures 3, 6). Whilst tonic regulation of EGFRwt has been previously described to involve phosphatases [23–26], such phenomenon has not been described for EGFRvIII. This suggests that constitutive activity of EGFRvIII, represented as elevated phosphorylation levels, is likely to be a combination of the kinase activity and regulation by phosphatases. Determination of the specific roles those two mechanisms play can have detrimental effects on the development of therapeutic approaches in the future.

In summary, we have shown that EGFRvIII trans-phosphorylates itself in homodimers in a continuous manner. This process is actively counteracted by a single or multiple tyrosine phosphatases, but not by protein degradation, which does not occur despite phosphorylation of key residues, suggesting steric hindrance. Results of a number of experiments investigating direct interaction as well as functional aspect of dimerization have indicated the wild-type receptor not to play a role in the process of EGFRvIII phosphorylation. Combined with the fact that this process is actively suppressed by EGFR TKIs, it can be concluded that EGFRvIII trans-phosphorylates itself in a continuous manner. The free cysteine at position 16 mediates formation of stable, covalent dimers, with small fraction of the EGFRvIII likely to dimerize transiently.

## MATERIALS AND METHODS

### Cell culture

DK-MG cells were purchased from Leibniz-Institut DSMZ (Braunschweig, Germany, Cat. no. ACC-277) and cultured in RPMI 1640 (GIBCO, Life Technologies, Grand Isle, NY, Cat. no. 11875-093). Obtaining of U87-MGvIII clone 4.12, DK-MG^high^ and DK-MG^low^ lines was described previously [28].

AD293 cells were purchased from Agilent Technologies (Santa Clara, CA, USA, Cat. no. 240085) and cultured in complete DMEM High Glucose (Biowest, France, Cat. no. L0102-500). Parental cell line was either transfected or transduced with lentiviral vectors carrying appropriate transgenes (prepared as described below) and positively selected using puromycin (5 μg/mL, InvivoGen, Cat. no. ant-pr-1).

The media used for culturing of cell lines were supplemented with 10% FBS (PAA, The Cell Culture Company, Austria, Cat. no. A15-104), 1% penicillin/streptomycin (Life Technologies, Carlsbad, CA, USA; Cat. no. 15140-122), 0.2% Gentamycin (Biowest, France, Cat. no. L0011-100). Cells were cultured under standard cell-culture conditions (5% CO_2_, 16% O_2_, 37°C).

Glioma cells were cultured as described previously [28]. Study was approved and conducted in accordance to the Approval No. RNN/234/17/KE from the Bioethical Committee of the Medical University of Lodz.

### Construction of plasmids and introduction of mutations into EGFRvIII transgene

Construction of the pLV1-puro-DEST plasmid and EGFRvIII insertion was performed as described previously [28, 53].

Site-directed mutagenesis was performed on pENTR/EGFRvIII by PCR using Q5 Hot Start High-Fidelity DNA Polymerase (New England Biolabs, Cat. no. M0493S), according to manufacturer guidelines. Primers used for generation of C16S - 5′CCTGTGGGGCCGAC AGCTATGA3′ and 5′CTCGGACGCTCGAGCCGTGA T3′; KD - 5′CGTCGCTATCGCCGAATTAAGAGAAG C3′ and 5′GGAATTTTAACTTTCTCACCTTCTGGG3′; to insert FLAG at the C-terminal - 5′GATGACGACAAG ACCGGTACCCAGCTTTCTTGTACAAAG3′ and 5′GTC TTTGTAGTCCTCGAGTGCTCCAATAAATTCACTGC 3′. Resulting linear plasmids were phosphorylated with T4PNK (NEB, Cat. no. M0201), ligated by T4 DNA Ligase (NEB, Cat. no. M0202) and purified by DpnI digest (NEB, Cat. no. R0176). Gateway LR Clonase II Enzyme mix (Life Technologies, Cat. no. 11791-020) was used to subclone the mutated EGFRvIII cassette to pLV1-puro-DEST or pIRESneo3-DEST vectors.

### Preparation of genetic content delivery vehicle and establishment of cell lines

Stable cell lines expressing EGFRvIII, EGFRvIIIKD, EGFRvIII-FLAG or EGFRvIII^C16S^ were established by transduction with lentivirus obtained using the LENTI Smart kit (InvivoGen, Cat. Code Its-str) as described previously [28]. HT-1080 cell line (ATCC, Cat. no. CCL-12) was used to titter lentiviral vectors based on puromycin resistance. The only exception was AD293vIII line, which was prepared by transfection of AD293 parental line with pIRESneo3-EGFRvIII using Fugene HD (Promega, Cat. no. E2311), selecting cells with neomycin treatment (500 µg/mL, InvivoGen, Cat. no. ANT-GN-1) and establishing monoclonal population, as described previously [53].

### Chemicals

To analyse the effects of pharmacological inhibitors, cells were seeded in 6-well plates at 2.5x10^5^ cells per well, incubated overnight to allow for adhesion and serum starved for subsequent 24 h. Serum free medium containing appropriate inhibitors was used to pre-treat cells for 30 min, unless indicated otherwise, prior to stimulation with 20 ng/mL EGF (Sigma-Aldrich, St Louis, MO, USA, Cat. no. E5036), 1 mM sodium orthovanadate – NaOVa (Na_3_VO_4_, Calbiochem, Cat. no. 567540), phenylarsine oxide – PAO (Sigma, Cat. no. P3075) or pervanadate – pV (obtained as described previously [22]; using Catalase, Sigma, Cat. no. C1345 and H_2_O_2_ Sigma, Cat. no. 31642) for 1 hour and lysed. Following concentrations of inhibitors were used: DMSO (Sigma-Aldrich, Cat. no. D2438, solvent control), 10 μM erlotinib (Tarceva, Molecula; Cat. no. 89983631), 0.5 μM afatinib (Selleckchem, Cat. no. S1011); 5 μM gefitinib (CellSignaling; 4765), 5 μM lapatinib (Selleckchem, Cat. no. S1028), 300 nM flavopiridol (Selleckchem, Cat. no. S1230), 5 μM arry380 (Gentaur Europe, Cat. no. A8366), 10 μM Nu7441 (Selleckchem, Cat. no. S2638) and 10 μM ruxolitinib (Selleckchem, Cat. no. S1378).

### Depletion of EGFRwt

The constructs carrying shRNA targeting EGFRwt or scrambled (SCR) control, were generated using the Block-iT U6 RNAi Entry vector kit (Life Technologies, Cat. no. K4945-00) and the following primers: WT-shRNA-F 5′CACCGTGAACCCCGAGGG CAAATACACGAATGTATTTGCCCTCGGGGTTCA3′; WT-shRNA-R 5′AAAATGAACCCCGAGGGCAAATA CATTCGTGTATTTGCCCTCGGGGTTCAC3′; Scr-shRNA-F 5′CACCGGGAGGTAAACGTAAGAGATAACGAATTA TCTCTTACGTTTACCTCC3′; Scr-shRNA-R 5′AAAA GGAGGTAAACGTAAGAGATAATTCGTTATCTCTTA CGTTTACCTCCC3′. Generated constructs were cloned into pLV1-puro-DEST vector using Gateway LR reaction, as described above. Plasmids were used to prepare lentiviral vectors and transiently transduce DK-MG cells. Knock down efficiency was determined on mRNA level by quantitative real-time RT-PCR, as described previously [3, 28].

As an alternative approach EGFRwt was depleted as a consequence of ligand binding. DK-MG cells were seeded in 6-well plates at 2.5 × 10^5^ cells per well, incubated overnight to allow for adhesion and serum starved for subsequent 24 h. Cells were pre-treated with cycloheximide (10 µg/mL; Sigma-Aldrich, Cat. no. 01810) for 30 min and stimulated with EGF for 3 h. Cells were then incubated for another 30 min with DMSO or gefitinib prior to 1 h treatment with NaOVa, fixed for immunocytochemistry or lysed for Western blotting.

### Western blotting

Denaturing blots were performed as described previously [28] using indicated antibodies (Table 1). To assess receptor dimerization, confluent AD293 cells were harvested using cell scraper, washed twice in DPBS and treated with appropriate compounds for one hour. Thereafter, cells were lysed for 30 min at 4°C in cell lysis buffer (50 mM Tris-HCl pH 7.5, 1 mM EDTA, 1 mM EGTA, 1 mM Sodium Orthovanadate, 10 µM β-glycerophosphate, 5 µM Sodium Pyrophosphate and 0.5% Triton X-100) freshly supplemented with Protease Inhibitor Cocktail (Sigma, Cat. no. P8340). Lysates were clarified at 7,000 × g for 5 min at 4°C. Equal protein amounts, as determined by BCA assay (Thermo Scientific, Cat. no. 23225), were mixed in reducing (with 1% beta-mercaptoethanol) or non-reducing Laemmli buffer and analysed by SDS-PAGE on 4-20% gradient precast gels (Mini-Protean TGX, Bio-Rad, Cat. no. 456-1096) and Western blotting. Densitometry measurements were performed in Quantity-One or ImageJ software.

**Table 1:** Antibodies used in the study

ANTIBODIES USED IN WESTERN BLOT			
Antibody	Host	Manufacturer	Dilution
anti-EGFR (1005)	rabbit	Santa Cruz Biotechnology, Inc., sc-03	1 : 200
anti-phospho-EGFR (Tyr992)	rabbit	Cell Signaling Technology, Inc., 2235	1 : 400
anti-phospho-EGFR (Tyr1045)	rabbit	Cell Signaling Technology, Inc., 2237	1 : 400
anti-phospho-EGFR (Tyr1068)	rabbit	Cell Signaling Technology, Inc., 2234	1 : 500
anti-phospho-EGFR (Tyr1148)	rabbit	Merck Millipore, 07-819	1 : 750
anti-phospho-EGFR (Tyr1173)	rabbit	Cell Signaling Technology, Inc., 4407	1 : 1000
anti-Actin, clone C4	mouse	Merck Millipore, MAB1501	1 : 3000
anti-FLAG M2	mouse	Sigma-Aldrich, F3165	1:1000
anti-phosphotyrosine	mouse	Calbiochem, 525295	1:200
anti-rabbit IgG-HRP	goat	Santa Cruz Biotechnology, Inc., sc-2004	1 : 4000
anti-mouse IgG-HRP	goat	Santa Cruz Biotechnology, Inc., sc-2005	1 : 4000

ANTIBODIES USED FOR IMMUNOFLUORESCENT STAINING			
Antibody	Host	Manufacturer	Dilution
anti-EGFR (1005)	rabbit	Santa Cruz Biotechnology, Inc., sc-03	1 : 250
anti-EGFR (R-1)	mouse	Santa Cruz Biotechnology, Inc., sc-101	1 : 50
anti-mouse Alexa Fluor^®^594	donkey	Molecular Probes, Invitrogen	1 : 500
anti-rabbit Alexa Fluor^®^488	donkey	Molecular Probes, Invitrogen	1 : 500

### Co-immunoprecipitation

Lysates prepared as for Western blotting were incubated for 4 h at 4°C with anti-FLAG M2 antibody (Sigma Aldrich, F3165) at 1:50 (v/v) dilution, with agitation. Subsequently, 1:50 of magnetic Protein G coated Dynabeads (Life Technologies, 10003D) was added and incubated over-night with agitation at 4°C. Beads were washed 6x with lysis buffer, boiled in reducing Laemmli buffer and supernatant was loaded onto gel for SDS-PAGE.

## SUPPLEMENTARY MATERIALS FIGURES AND TABLE



## References

[1] Friedman HS, Kerby T, Calvert H (2000). Temozolomide and treatment of malignant glioma. Clin Cancer Res.

[2] Yamazaki H, Ohba Y, Tamaoki N, Shibuya M (1990). A deletion mutation within the ligand binding domain is responsible for activation of epidermal growth factor receptor gene in human brain tumors. Jpn J Cancer Res.

[3] Peciak J, Stec WJ, Treda C, Ksiazkiewicz M, Janik K, Popeda M, Smolarz M, Rosiak K, Hulas-Bigoszewska K, Och W, Rieske P, Stoczynska-Fidelus E (2017). Low Incidence along with Low mRNA Levels of EGFRvIII in Prostate and Colorectal Cancers Compared to Glioblastoma. J Cancer.

[4] Grandal MV, Zandi R, Pedersen MW, Willumsen BM, van Deurs B, Poulsen HS (2007). EGFRvIII escapes down-regulation due to impaired internalization and sorting to lysosomes. Carcinogenesis.

[5] Huang HS, Nagane M, Klingbeil CK, Lin H, Nishikawa R, Ji XD, Huang CM, Gill GN, Wiley HS, Cavenee WK (1997). The enhanced tumorigenic activity of a mutant epidermal growth factor receptor common in human cancers is mediated by threshold levels of constitutive tyrosine phosphorylation and unattenuated signaling. J Biol Chem.

[6] Schmidt MH, Furnari FB, Cavenee WK, Bögler O (2003). Epidermal growth factor receptor signaling intensity determines intracellular protein interactions, ubiquitination, and internalization. Proc Natl Acad Sci USA.

[7] Davies GC, Ryan PE, Rahman L, Zajac-Kaye M, Lipkowitz S (2006). EGFRvIII undergoes activation-dependent downregulation mediated by the Cbl proteins. Oncogene.

[8] Han W, Zhang T, Yu H, Foulke JG, Tang CK (2006). Hypophosphorylation of residue Y1045 leads to defective downregulation of EGFRvIII. Cancer Biol Ther.

[9] Chu CT, Everiss KD, Wikstrand CJ, Batra SK, Kung HJ, Bigner DD (1997). Receptor dimerization is not a factor in the signalling activity of a transforming variant epidermal growth factor receptor (EGFRvIII). Biochem J.

[10] Hwang Y, Chumbalkar V, Latha K, Bogler O (2011). Forced dimerization increases the activity of ΔEGFR/EGFRvIII and enhances its oncogenicity. Mol Cancer Res.

[11] Kancha RK, von Bubnoff N, Duyster J (2013). Asymmetric kinase dimer formation is crucial for the activation of oncogenic EGFRvIII but not for ERBB3 phosphorylation. Cell Commun Signal.

[12] Fernandes H, Cohen S, Bishayee S (2001). Glycosylation-induced conformational modification positively regulates receptor-receptor association: a study with an aberrant epidermal growth factor receptor (EGFRvIII/DeltaEGFR) expressed in cancer cells. J Biol Chem.

[13] Gajadhar AS, Bogdanovic E, Muñoz DM, Guha A (2012). In situ analysis of mutant EGFRs prevalent in glioblastoma multiforme reveals aberrant dimerization, activation, and differential response to anti-EGFR targeted therapy. Mol Cancer Res.

[14] Luwor RB, Zhu HJ, Walker F, Vitali AA, Perera RM, Burgess AW, Scott AM, Johns TG (2004). The tumor-specific de2-7 epidermal growth factor receptor (EGFR) promotes cells survival and heterodimerizes with the wild-type EGFR. Oncogene.

[15] Ymer SI, Greenall SA, Cvrljevic A, Cao DX, Donoghue JF, Epa VC, Scott AM, Adams TE, Johns TG, Greenall SA, Cvrljevic A, Cao DX, Donoghue JF (2011). Glioma specific extracellular missense mutations in the first cysteine rich region of epidermal growth factor receptor (EGFR) initiate ligand independent activation. Cancers (Basel).

[16] Fan QW, Cheng CK, Gustafson WC, Charron E, Zipper P, Wong RA, Chen J, Lau J, Knobbe-Thomsen C, Weller M, Jura N, Reifenberger G, Shokat KM, Weiss WA (2013). EGFR phosphorylates tumor-derived EGFRvIII driving STAT3/5 and progression in glioblastoma. Cancer Cell.

[17] Greenall SA, Donoghue JF, Van Sinderen M, Dubljevic V, Budiman S, Devlin M, Street I, Adams TE, Johns TG (2015). EGFRvIII-mediated transactivation of receptor tyrosine kinases in glioma: mechanism and therapeutic implications. Oncogene.

[18] Li L, Puliyappadamba VT, Chakraborty S, Rehman A, Vemireddy V, Saha D, Souza RF, Hatanpaa KJ, Koduru P, Burma S, Boothman DA, Habib AA (2015). EGFR wild type antagonizes EGFRvIII-mediated activation of Met in glioblastoma. Oncogene.

[19] Fromm JA, Johnson SA, Johnson DL (2008). Epidermal growth factor receptor 1 (EGFR1) and its variant EGFRvIII regulate TATA-binding protein expression through distinct pathways. Mol Cell Biol.

[20] Gan HK, Cvrljevic AN, Johns TG (2013). The epidermal growth factor receptor variant III (EGFRvIII): where wild things are altered. FEBS J.

[21] Nishikawa R, Ji XD, Harmon RC, Lazar CS, Gill GN, Cavenee WK, Huang HJ (1994). A mutant epidermal growth factor receptor common in human glioma confers enhanced tumorigenicity. Proc Natl Acad Sci USA.

[22] Huyer G, Liu S, Kelly J, Moffat J, Payette P, Kennedy B, Tsaprailis G, Gresser MJ, Ramachandran C (1997). Mechanism of inhibition of protein-tyrosine phosphatases by vanadate and pervanadate. J Biol Chem.

[23] Reynolds AR, Tischer C, Verveer PJ, Rocks O, Bastiaens PI (2003). EGFR activation coupled to inhibition of tyrosine phosphatases causes lateral signal propagation. Nat Cell Biol.

[24] Offterdinger M, Georget V, Girod A, Bastiaens PI (2004). Imaging phosphorylation dynamics of the epidermal growth factor receptor. J Biol Chem.

[25] Monast CS, Furcht CM, Lazzara MJ (2012). Computational analysis of the regulation of EGFR by protein tyrosine phosphatases. Biophys J.

[26] Kleiman LB, Maiwald T, Conzelmann H, Lauffenburger DA, Sorger PK (2011). Rapid phospho-turnover by receptor tyrosine kinases impacts downstream signaling and drug binding. Mol Cell.

[27] Ruff SJ, Chen K, Cohen S (1997). Peroxovanadate induces tyrosine phosphorylation of multiple signaling proteins in mouse liver and kidney. J Biol Chem.

[28] Stec WJ, Rosiak K, Siejka P, Peciak J, Popeda M, Banaszczyk M, Pawlowska R, Treda C, Hulas-Bigoszewska K, Piaskowski S, Stoczynska-Fidelus E, Rieske P (2016). Cell line with endogenous EGFRvIII expression is a suitable model for research and drug development purposes. Oncotarget.

[29] Stockhausen MT, Kristoffersen K, Stobbe L, Poulsen HS (2014). Differentiation of glioblastoma multiforme stem-like cells leads to downregulation of EGFR and EGFRvIII and decreased tumorigenic and stem-like cell potential. Cancer Biol Ther.

[30] Needham SR, Roberts SK, Arkhipov A, Mysore VP, Tynan CJ, Zanetti-Domingues LC, Kim ET, Losasso V, Korovesis D, Hirsch M, Rolfe DJ, Clarke DT, Winn MD (2016). EGFR oligomerization organizes kinase-active dimers into competent signalling platforms. Nat Commun.

[31] Fabian MA, Biggs WH, Treiber DK, Atteridge CE, Azimioara MD, Benedetti MG, Carter TA, Ciceri P, Edeen PT, Floyd M, Ford JM, Galvin M, Gerlach JL (2005). A small molecule-kinase interaction map for clinical kinase inhibitors. Nat Biotechnol.

[32] Park JH, Liu Y, Lemmon MA, Radhakrishnan R (2012). Erlotinib binds both inactive and active conformations of the EGFR tyrosine kinase domain. Biochem J.

[33] Liccardi G, Hartley JA, Hochhauser D (2011). EGFR nuclear translocation modulates DNA repair following cisplatin and ionizing radiation treatment. Cancer Res.

[34] Dowlati A, Nethery D, Kern JA (2004). Combined inhibition of epidermal growth factor receptor and JAK/STAT pathways results in greater growth inhibition *in vitro* than single agent therapy. Mol Cancer Ther.

[35] Narita Y, Nagane M, Mishima K, Huang HJ, Furnari FB, Cavenee WK (2002). Mutant epidermal growth factor receptor signaling down-regulates p27 through activation of the phosphatidylinositol 3-kinase/Akt pathway in glioblastomas. Cancer Res.

[36] Muhsin M, Graham J, Kirkpatrick P (2003). Fresh from the pipeline: Gefitinib. Nat Rev Drug Discov.

[37] Wiley HS (2003). Trafficking of the ErbB receptors and its influence on signaling. Exp Cell Res.

[38] Huang F, Goh LK, Sorkin A (2007). EGF receptor ubiquitination is not necessary for its internalization. Proc Natl Acad Sci USA.

[39] Opresko LK, Chang CP, Will BH, Burke PM, Gill GN, Wiley HS (1995). Endocytosis and lysosomal targeting of epidermal growth factor receptors are mediated by distinct sequences independent of the tyrosine kinase domain. J Biol Chem.

[40] Macdonald-Obermann JL, Piwnica-Worms D, Pike LJ (2012). Mechanics of EGF receptor/ErbB2 kinase activation revealed by luciferase fragment complementation imaging. Proc Natl Acad Sci U S A.

[41] Coban O, Zanetti-Dominguez LC, Matthews DR, Rolfe DJ, Weitsman G, Barber PR, Barbeau J, Devauges V, Kampmeier F, Winn M, Vojnovic B, Parker PJ, Lidke KA (2015). Effect of phosphorylation on EGFR dimer stability probed by single-molecule dynamics and FRET/FLIM. Biophys J.

[42] Lu C, Mi LZ, Schürpf T, Walz T, Springer TA (2012). Mechanisms for kinase-mediated dimerization of the epidermal growth factor receptor. J Biol Chem.

[43] Greenall SA, Donoghue JF, Gottardo NG, Johns TG, Adams TE (2015). Glioma-specific Domain IV EGFR cysteine mutations promote ligand-induced covalent receptor dimerization and display enhanced sensitivity to dacomitinib *in vivo*. Oncogene.

[44] Li L, Chakraborty S, Yang CR, Hatanpaa KJ, Cipher DJ, Puliyappadamba VT, Rehman A, Jiwani AJ, Mickey B, Madden C, Raisanen J, Burma S, Saha D (2014). An EGFR wild type-EGFRvIII-HB-EGF feed-forward loop regulates the activation of EGFRvIII. Oncogene.

[45] Ymer SI, Greenall SA, Cvrljevic A, Cao DX, Donoghue JF, Epa VC, Scott AM, Adams TE, Johns TG (2011). Glioma Specific Extracellular Missense Mutations in the First Cysteine Rich Region of Epidermal Growth Factor Receptor (EGFR) Initiate Ligand Independent Activation. Cancers (Basel).

[46] Kluba M, Engelborghs Y, Hofkens J, Mizuno H (2015). Inhibition of Receptor Dimerization as a Novel Negative Feedback Mechanism of EGFR Signaling. PLoS One.

[47] Hwang Y, Chumbalkar V, Latha K, Bogler O (2011). Forced dimerization increases the activity of ΔEGFR/EGFRvIII and enhances its oncogenicity. Mol Cancer Res.

[48] Ceresa BP, Vanlandingham PA (2008). Molecular Mechanisms that Regulate Epidermal Growth Factor Receptor Inactivation. Clin Med Oncol.

[49] Sorkin A (2001). Internalization of the epidermal growth factor receptor: role in signalling. Biochem Soc Trans.

[50] Platta HW, Stenmark H (2011). Endocytosis and signaling. Curr Opin Cell Biol.

[51] Ramnarain DB, Park S, Lee DY, Hatanpaa KJ, Scoggin SO, Otu H, Libermann TA, Raisanen JM, Ashfaq R, Wong ET, Wu J, Elliott R, Habib AA (2006). Differential gene expression analysis reveals generation of an autocrine loop by a mutant epidermal growth factor receptor in glioma cells. Cancer Res.

[52] Tang P, Steck PA, Yung WK (1997). The autocrine loop of TGF-α/EGFR and brain tumors. J Neurooncol.

[53] Treda C, Popeda M, Ksiazkiewicz M, Grzela DP, Walczak MP, Banaszczyk M, Peciak J, Stoczynska-Fidelus E, Rieske P (2016). EGFR Activation Leads to Cell Death Independent of PI3K/AKT/mTOR in an AD293 Cell Line. PLoS One.

