# Supplementary material for: Cyclic trans-phosphorylation in a homodimer as the predominant mechanism of EGFRvIII action and regulation
Source: Oncotarget. 2018 Jan 6;9(9):8560–72. doi: 10.18632/oncotarget.24058 (PMC5823601; doi:10.18632/oncotarget.24058)
Supplement: Supplementary file 1 [file oncotarget-09-8560-s001.pdf]

Cyclic *trans*-phosphorylation in a homodimer as the predominant mechanism of EGFRvIII action and regulation

SUPPLEMENTARY MATERIALS

Immunocytochemistry

Performed as described previously [1] using indicated antibodies (Table 1). Images were processed using ImageJ software [2].

Real-time RT-PCR

Performed as described previously [3]. Supplementary Table 1 summarizes primer sequences used. The pool for qRT-PCR containing EGFRvIII, which does not occur in normal tissue, was obtained from mRNA isolated from 15 cancer tissues positive for mutant receptor as described in [4]. All cycle threshold values were normalised to TBP levels using the Pfaffl method [5].

REFERENCES

1. Stoczynska-Fidelus E, Piaskowski S, Bienkowski M, Banaszczyk M, Hulas-Bigoszewska K, Winiecka-Klimek M, Radomiak-Zaluska A, Och W, Borowiec M, Zieba J, Treda C, Rieske P. The failure in the stabilization of glioblastoma-derived cell lines: spontaneous *in vitro* senescence as the main culprit. PLoS One. 2014; 9:e87136.
2. Schneider CA, Rasband WS, Eliceiri KW. NIH Image to ImageJ: 25 years of image analysis. Nat Methods. 2012; 9:671–75.
3. Zieba J, Ksiazkiewcz M, Janik K, Banaszczyk M, Peciak J, Piaskowski S, Lipinski M, Olczak M, Stoczynska-Fidelus E, Rieske P. Sensitivity of neoplastic cells to senescence unveiled under standard cell culture conditions. Anticancer Res. 2015; 35:2759–68.
4. Stec WJ, Rosiak K, Siejka P, Peciak J, Popeda M, Banaszczyk M, Pawlowska R, Treda C, Hulas-Bigoszewska K, Piaskowski S, Stoczynska-Fidelus E, Rieske P. Cell line with endogenous EGFRvIII expression is a suitable model for research and drug development purposes. Oncotarget. 2016; 7:31907–25. https://doi.org/10.18632/oncotarget.8201.
5. Pfaffl MW. A new mathematical model for relative quantification in real-time RT-PCR. Nucleic Acids Res. 2001; 29:e45.

Supplementary Table 1: Primer sequences used for RT-PCR

Primer	Sequence
TBP_F	5′ GAGCTGTGATGTGAAGTTTCC 3′
TBP_R	5′ TCTGGGTTTGATCATTCTGTAG 3′
EGFRvIII_F	5′ GGCTCTGGAGGAAAAGAAAGGTAATTATGT 3′
EGFRvIII_R	5′ ACCAATACCTATTCCGTTACACACT 3′
EGFRwt_F	5′ TAGCAGTCTTATCTAACTATGAT 3′
EGFRwt_R	5′ CACTGCTGACTATGTCCCGC 3′

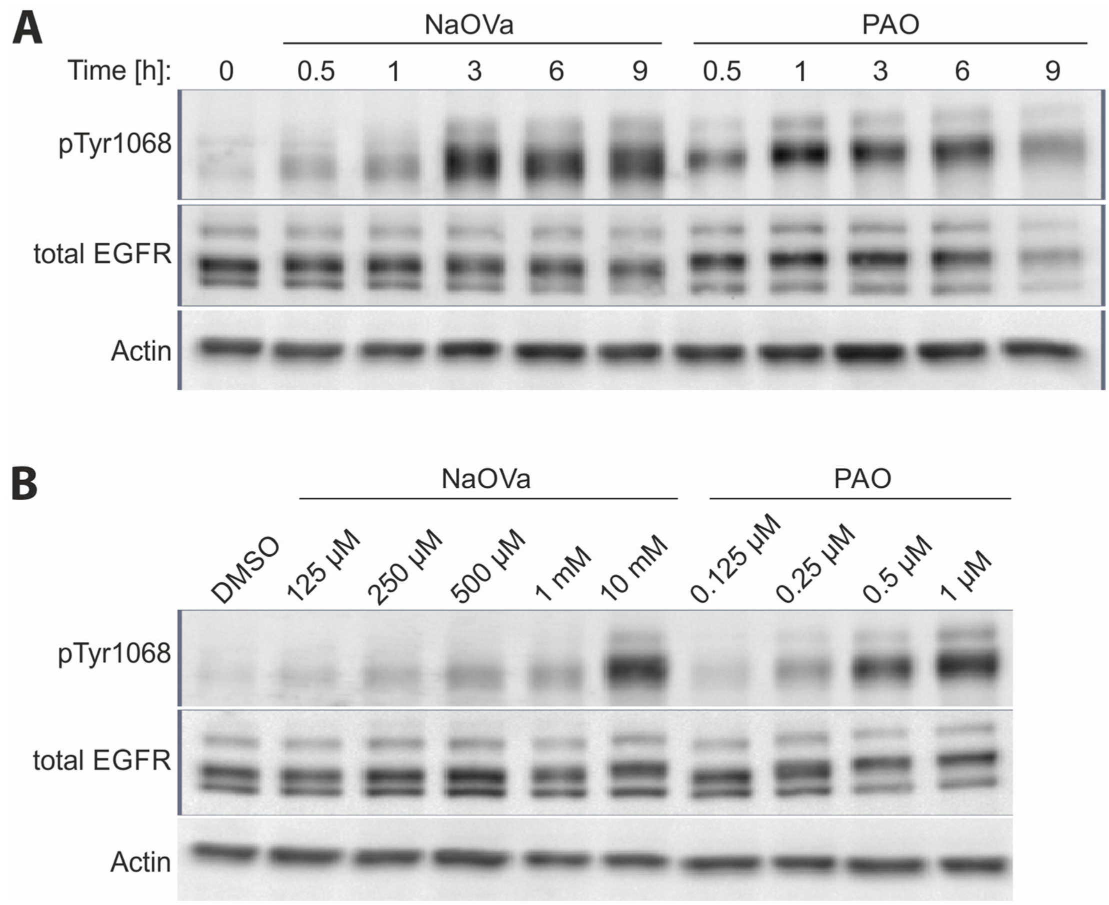

Supplementary Figure 1: Assessment of time- and dose-dependence of PTP inhibitors treatment on EGFR phosphorylation. (**A**) DK-MG[high] cells were treated with 1mM NaOVa or 0.5 μM PAO for indicated period of time, prior to lysis. Cell viability was decreased after 9 hours of NaOVa treatment and 3 hours of PAO treatment. Experiment was conducted in the presence of cycloheximide to prevent *de novo* protein synthesis. (**B**) Cells were treated with increasing concentration of NaOVa or PAO as indicated, for 1 hour. Viability of cells was decreased above 1 mM of NaOVa and 1μM PAO.

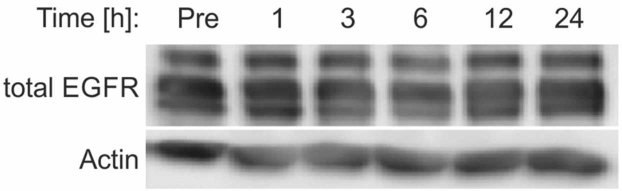

Supplementary Figure 2: EGFRvIII is stable in the DK-MG cell line. Cells treated with cycloheximide were incubated in serum-free medium and lysed at indicated time-points to be resolved by SDS-PAGE.

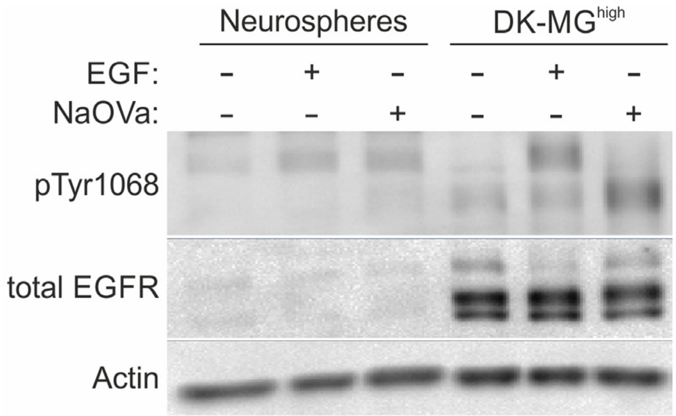

Supplementary Figure 3: Phosphorylation profile of EGFRvIII in primary glioma cells is similar to that observed in DK-MG cell line. Primary cells were obtained from collagenase/dispase treated resected tumor mass and cultured as neurospheres, which were treated with 20 ng/ml EGF or NaOVa for 1 h, prior to lysis and analysis by Western blotting.

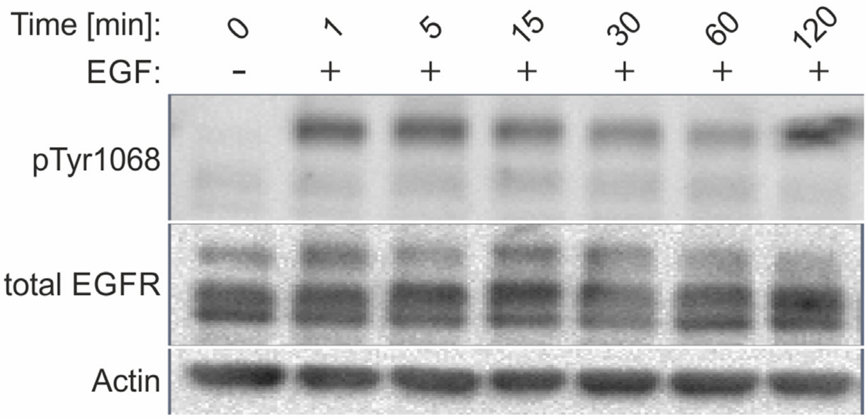

Supplementary Figure 4: Prolonged stimulation with EGF does not affect phosphorylation of EGFRvIII. DK-MG cells were treated with 20 ng/ml EGF and lysed at indicated time-points.

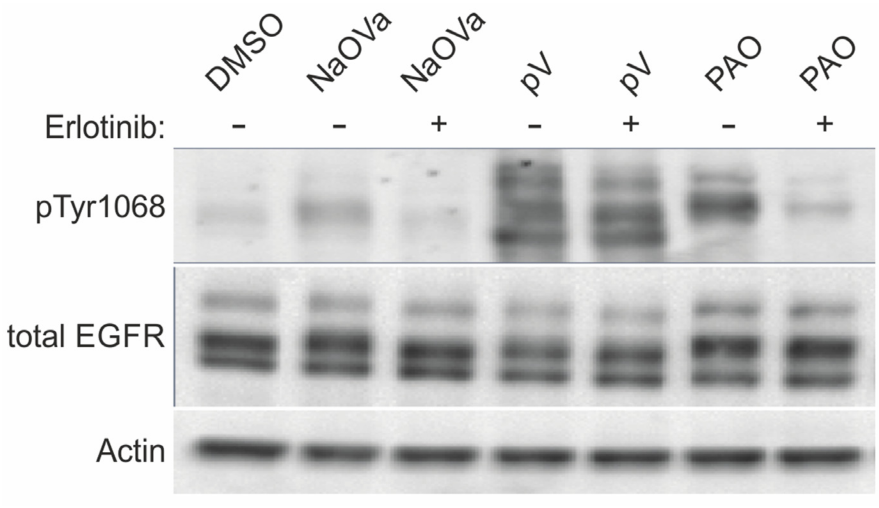

Supplementary Figure 5: Erlotinib inhibits phosphorylation resulting from PTP inhibitor treatment. DK-MG cells pre-treated for 15 min with 10 µM erlotinib were treated with 1 mM NaOVa, 10 µM pV and 0.5 µM PAO for 1 hour, lysed and analysed by Western blotting.

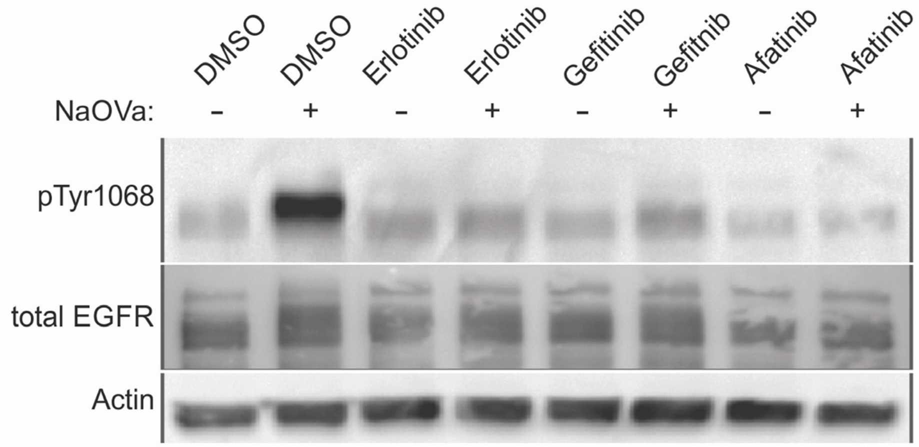

Supplementary Figure 6: EGFRvIII is hyperphosphorylated in U87MG in response to NaOVa treatment. Cells pre-treated with indicated EGFR kinase inhibitors were treated with NaOVa, lysed and resolved by Western blotting.

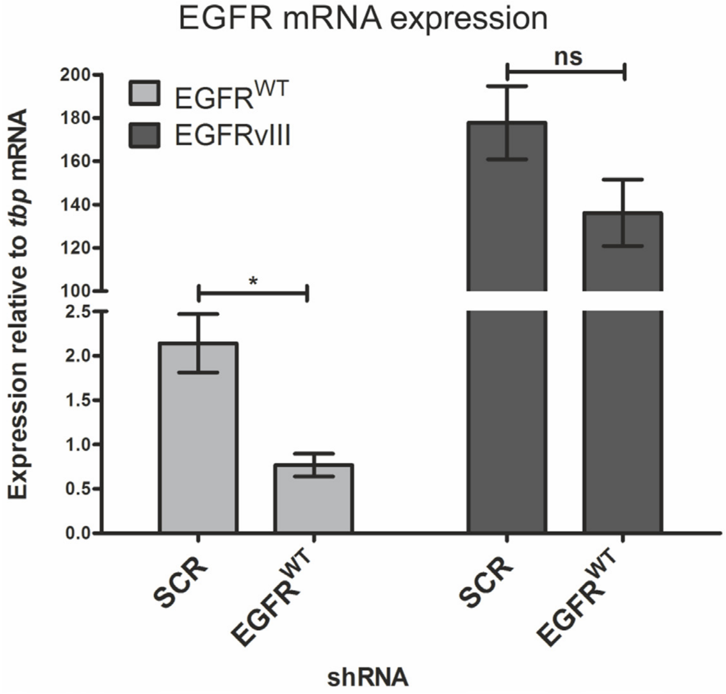

Supplementary Figure 7: Effectiveness of *EGFRwt* gene silencing. Cells transduced with control (SCR) shRNA or shRNA against the wild-type receptor were tested for effectiveness on the mRNA level. Statistical significance was calculated by two-tailed Student's *t*-test; $^{*}p < 0.05$; $^{**}p < 0.01$; ns – not significant. Error bars indicate SEM.

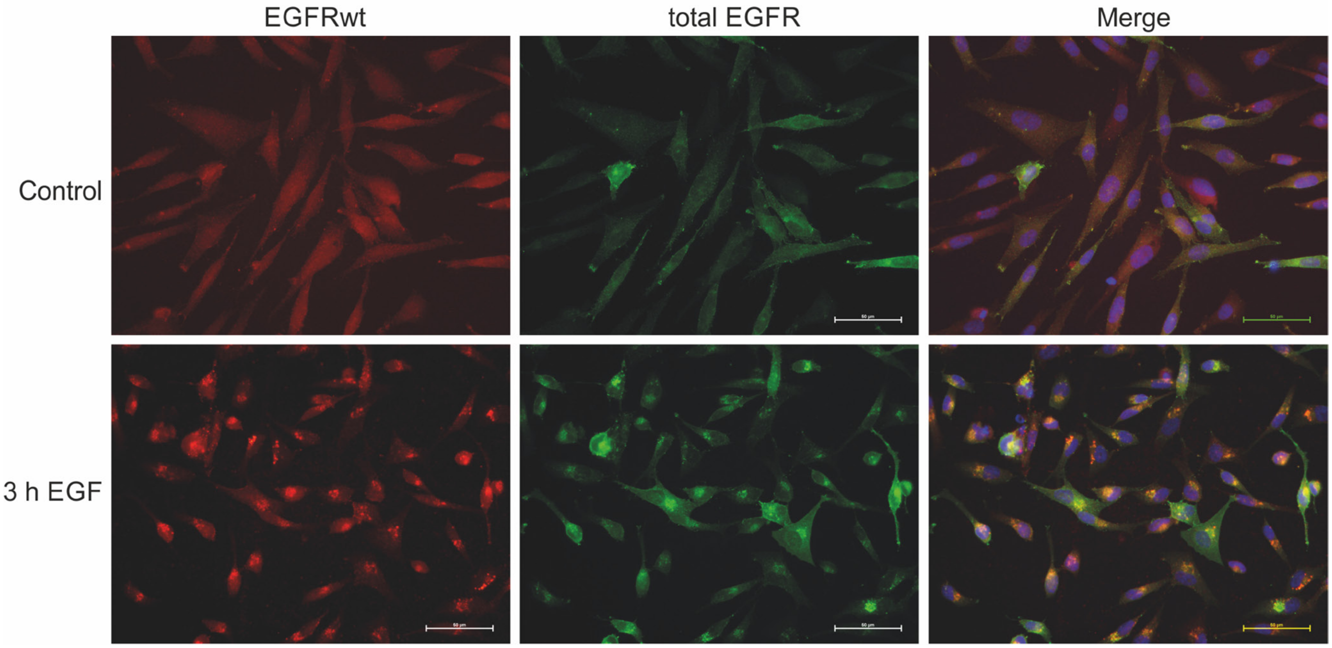

Supplementary Figure 8: Prolonged treatment with EGF causes internalization of the EGFRwt. DK-MG cells were concurrently treated for 3 h with EGF and cycloheximide to block *de novo* protein production. Intracellular dots are reminiscent of protein accumulated in the endocytic pathway organelles prior to degradation. Cells that remain uniformely positive for total EGFR following EGF stimulation strongly express EGFRvIII. Scale bar indicates 50 µm.

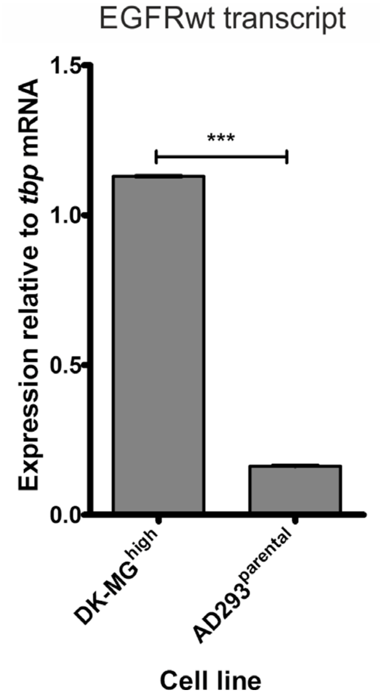

Supplementary Figure 9: AD293 cell line expresses EGFRwt to lower extent than DK-MG cell line does. Analysis of the mRNA levels of the EGF receptor in two cell lines. Statistical significance was calculated by two-tailed Student's *t*-test; $^{***}p < 0.005$. Error bars indicate SEM.

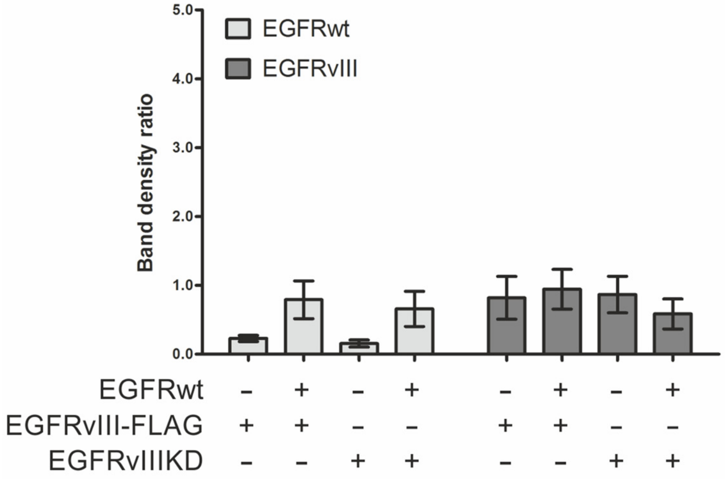

Supplementary Figure 10: Total protein levels of constructs transduced into AD293 cell line. Quantification of three Western blots of cells expressing indicated constructs. Total EGFR levels were normalized to actin. Error bars indicate SEM.

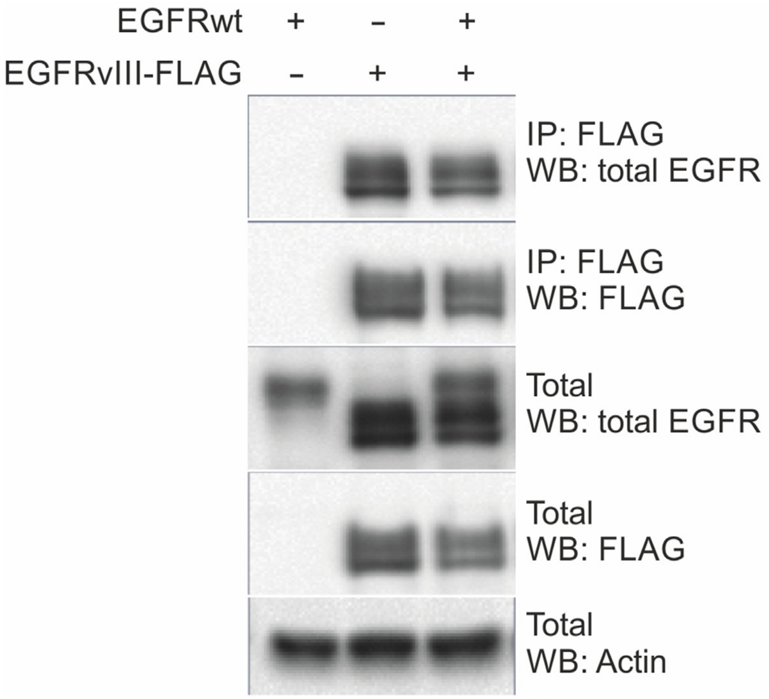

Supplementary Figure 11: No physical interaction between EGFRvIII and EGFRwt was detected. Co-immunoprecipitation analysis using lysates from cells expressing EGFRvIII-FLAG, EGFRwt or co-expressing EGFRwt with EGFRvIII-FLAG.
